# Botulinum Neurotoxin Type A in the Treatment of Facial Seborrhea and Acne: Evidence and a Proposed Mechanism

**DOI:** 10.3390/toxins13110817

**Published:** 2021-11-19

**Authors:** Nark-Kyoung Rho, Young-Chun Gil

**Affiliations:** 1 Department of Dermatology, Sungkyunkwan University School of Medicine, Seoul 06355, Korea; rhonark@hanmail.net; 2 Leaders Aesthetic Laser & Cosmetic Surgery Center, Seoul 06014, Korea; 3 Department of Anatomy, Chungbuk National University School of Medicine, Cheongju 28644, Korea

**Keywords:** acetylcholine, acne vulgaris, botulinum toxins, cholinergic receptors, non-neuronal cholinergic system, oily skin, sebaceous glands, seborrhea, sebum

## Abstract

Intradermal injection of botulinum neurotoxin is a frequently performed procedure in aesthetic dermatology to improve facial skin tone, texture, fine wrinkles, and enlarged pores. In practice, botulinum neurotoxin type A is also used to reduce skin oiliness of the face. There is increasing evidence that acetylcholine plays specific roles in sebum production, suggesting that botulinum neurotoxin type A may reduce sebum production by interfering with cholinergic transmission between sebaceous glands and autonomic nerve terminals. Botulinum neurotoxins can also inhibit several pathogenetic components of acne development, suggesting that botulinum neurotoxins can be used as a safe and effective treatment modality for acne and other skin disorders related to overactivity of sebaceous glands. This review aims to explore the current evidence behind the treatment of facial seborrhea and acne with botulinum neurotoxin type A.

## 1. Introduction

**Figure 1:**
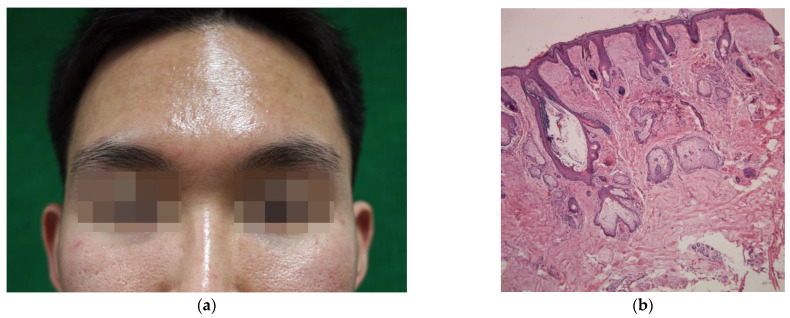
(**a**) A young Korean male with facial seborrhea appearing greasy and shiny, accompanied by enlarged pores on the cheeks; (**b**) Histologic findings of the facial skin of excess sebum secretion. Note the lobules of mature sebaceous glands and a dilated follicular infundibulum filled with keratinous material (nose, hematoxylin, and eosin staining, ×50).

A sebaceous gland (SG) is a microscopic exocrine gland in the skin that opens into a hair follicle to secrete an oily or waxy matter called sebum. Sebum production is physiologic and serves to lubricate the hairs and the stratum corneum. However, when produced in excess, it can be an aesthetic concern for many people since it frequently accompanies greasy skin and enlargement of facial pores (Figure 1). The consequences of excess sebum may be associated with adverse psychological and social effects resulting from skin oiliness and shine and prominent pores [1]. Facial seborrhea (“oily skin”), caused by an excessive sebum production in the facial skin, is also a major pathogenetic factor in several dermatologic disorders such as acne [2,3]. One of the most effective inhibitors of sebum production is oral isotretinoin. However, many patients cannot tolerate, are unwilling to accept the side effects, are contraindicated for its use, or do not have severe enough disease to justify its use [2]. The studies show that lasers and other energy-based treatments can reduce sebum production. However, a shorter duration of efficacy, potential side effects, and the relative lack of evidence are common drawbacks to these treatments.

Botulinum neurotoxin type A (BoNTA) blocks the release of acetylcholine (Ach) into the synaptic cleft, where it binds to a cholinergic receptor on a post-synaptic cell. Acting at the neuromuscular junction, BoNTA leads to a loss of muscle tone, while in some glandular tissues, it inhibits cholinergic sympathetic nerve function. The ability of BoNTA to inhibit cholinergic transmission prompted further investigations into its clinical use in several autonomic disorders resulting in glandular hypersecretion, such as hyperhidrosis and sialorrhea [1,2]. Another well-known cutaneous exocrine gland is the SG, opening into a hair follicle to secrete sebum. The neuronal control over the SG has long been implicated by clinical observations [4,5,6,7]. Empirical reports on the use of BoNTA to suppress excessive sebum suggest that BoNTA could modulate the neuroendocrine control over the SGs [8]. This review summarizes in vitro and in vivo studies relevant to BoNTA therapy for SG overactivity. We also reviewed the reported cases and clinical trials on the use of BoNTA for facial seborrhea, wide pores, and associated acne.

## 2. Search Strategy

To identify relevant reports and investigations to the BoNTA for seborrhea and acne, we performed a literature search in the following electronic databases: Cochrane Library, EBSCO, Google Scholar, NCBI, OVID, and PUBMED. A manual search in the list of references of the articles was also performed. The search aimed to find studies focused on the effect of BoNTA in facial sebum production and excretion. The main keywords were: (1) abobotulinumtoxinA OR Botox OR botulinum neurotoxin OR botulinum toxin OR Dysport OR incobotulinumtoxinA OR onabotulinumtoxinA OR Xeomin. (2) Acne OR oily skin OR sebaceous gland OR sebocyte OR seborrhea OR sebum. (3) 1 AND 2. Our search identified 75 records after removing duplicates. After examining the full text, 14 citations were excluded. To reflect specific points based on the authors’ experience, especially in clinical manifestations and histopathologic findings on which the citations are missing, representative clinical photographs and histopathologic images were searched in the medical records of the authors’ patients.

## 3. Botulinum Neurotoxin for Sebum Reduction

### 3.1. Anecdotal Clinical Reports

Unlike sweat secretion, sebum production has not been thought to be controlled by the autonomic nervous system [9]. It is often expressed as saying that “no nerve supply but only hormonal regulation” affects the sebaceous gland activity. Dermatological and endocrinological studies suggest that humoral mechanisms may control gland activity, and now it is widely accepted that androgenic hormones are the primary regulators responsible for sebum excretion [9]. However, clinical observations suggested a neuronal control over the human SG. In patients with partial facial paralysis, variations in the sebum secretion rate were observed between the unaffected and neurologically impaired halves of the face [4]. Unilateral facial acne was also observed following partial facial paralysis [5]. Sebum secretion from the thighs of paraplegic patients is significantly higher than that in non-paraplegic controls, while the sebum excretion rate is unchanged above the level of spinal injury [4]. Studies also showed that the use of topical anticholinergic agents resulted in a significant decrease in sebum secretion [7]. These clinical findings prompted many researchers to take a closer look at the relationship between the SG and cholinergic system, with multiple studies demonstrating the expression of ACh receptors on the SG [10,11].

### 3.2. Extraneuronal Cholinergic System of Sebaceous Glands

**Figure 2:**
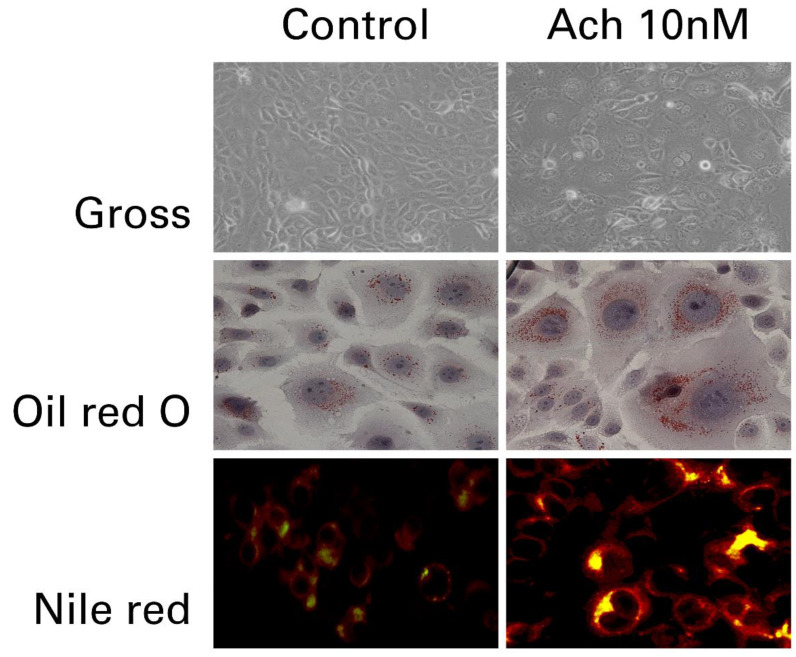
Increased lipid synthesis in cultured sebocytes after treatment with 10 nM of acetylcholine. Intracellular lipids are detected in acetylcholine-treated sebocytes by lipid staining (Photos are used with the kind permission of Dr. Myung Im, MD, IM Dermatology Clinic, Daejeon, Korea).

ACh acts through two principal receptor types, muscarinic and nicotonic [12]. The muscarinic receptor m2AChR is expressed in suprabasal sebocytes, and its signaling pathways in the SGs are well-described [13,14,15]. In comparison, ductal cells of the SG differentially show the highest immunoreactivity for or alpha 7 nicotinic acetylcholine receptor (α7nAChR), a major nicotinic ACh receptor subunit [10,16,17]. The α7nAChR is expressed in many non-neuronal tissues such as skin, although it was initially described as a neuronal receptor subtype [18]. The existence of non-neuronal cholinergic receptors in the SG suggests that nicotinic signaling pathways and ACh contribute to the pathophysiological processes in the pilosebaceous unit [18,19]. In both cultured sebocytes and healthy volunteers, ACh dose-dependently increased the lipid synthesis (Figure 2) by its interaction with α7nAChR via activation of ERK signaling [19], probably through promoting sebocyte differentiation [20]. It aligns with an immunohistochemical finding that mature sebocytes, in particular, show high positivity for α7nAChR [10]. In vitro, sebocytes from facial seborrhea are phenotypically fully mature and express more cholinergic receptors [19]. These findings suggest that ACh and its receptors play specific roles in sebocyte differentiation and sebum production [10,19,20], and it is plausible to assume that facial seborrhea may be more susceptible to cholinergic regulation than normal skin [19,21]. It remains to be seen how the human SG in vivo responds to the cholinergic stimuli in vivo. It has not been elucidated whether high doses of or prolonged exposure to Ach leads to depolarizing and desensitizing block of cholinergic receptor neurotransmission.

### 3.3. Early Observations

More than 40 years ago, researchers found that anticholinergics block cholinergic signaling and suppress ACh-induced sebum synthesis [19,21]. In a clinical trial involving ten patients with mild acne, a long-term application of a topical poldine methylmethosulphate, an anticholinergic agent, to the forehead skin produced a significant reduction in sebum excretion [7]. A marked decrease in sebum production and pore size was noted after facial seborrhea treatment with α-bungarotoxin, a well-known antagonist of the ACh receptor [18,22]. Therefore, it is not strange to expect that BoNTA, a potent inhibitor of ACh release from cholinergic nerves, may have a suppressive effect on the SG. An animal study demonstrated a reduction in the number of sebocytes after BoNTA injection [23]. Clinically, anecdotal reports describe a decrease in facial sebum secretion after injection of BoNTA. Regression of the sebaceous cyst was described after BoNTA injection for the treatment of migraine [24]. Jankovic [25] suggested that facial seborrhea in Parkinson’s disease may respond to botulinum neurotoxins. The prevalence of local skin dryness, suggestive of reduced sebum secretion, was 8% in one clinical study to evaluate the safety and efficacy of BoNTA injection to treat forehead wrinkles [26]. Wu [27] described a decreased sebum production after intradermal injection of BoNTA for cosmetic purposes. These laboratory findings and clinical observations raised speculation that BoNTA may be an effective treatment for excessive sebum production, which prompted further clinical trials.

### 3.4. OnabotulinumtoxinA

A report by Shah [28] was one of the early clinical trials that demonstrated a favorable result of BoNTA in reducing sebum production and pore size. In this retrospective study, twenty subjects with facial seborrhea and enlarged pores were treated using intradermal injection of onabotulinumtoxinA (Botox; Allergan, CA, USA) to the “T-zone” of the face and photographically evaluated one month after treatment. Seventeen of 20 subjects showed improvement in oiliness after one treatment, with no significant complications. This study was quoted as the first step towards a more profound understanding of BoNTA actions on the sebaceous glands. However, the lack of objective measurements strongly limits the study since the author used a photographic assessment to measure sebum production and pore size, as criticized by other researchers that the decrease in oiliness and shininess noted in the photograph may represent a decrease in sweating [2]. This study addresses the need for objective evaluation to quantitatively assess the decrease in sebum production and exclude confounding factors.

Sebumeter is a device that quantifies the sebum amount from the skin in an objective manner. A photoelectric receiver measures the amount of light passing through the sebum-covered translucent element and calculates the result [29]. Several researchers used this device to objectively measure the sebum production before and after BoNTA injections (Table 1). In a study involving 20 patients with facial seborrhea, the researchers used a sebumeter to measure the change in sebum production after intradermal injection of onabotulinumtoxinA [30]. There was an average decrease in sebum production of 36.2% at two weeks, 29.9% at four weeks, and 25.6% at six weeks follow-up, with patients’ satisfaction up to 3 months. In another clinical trial of 15 females who were injected with BoNTA in glabella and forehead muscles, sebumeter readings showed a maximum decrease in sebum production (26.8%) four weeks after treatment and returned to baseline after 16 weeks. Another group of investigators reported a 24.3% decrease in skin surface lipid at two weeks after intradermal injection of BoNTA in 10 patients with acne [31], similar to previous studies. It should be pointed out that the clinical improvement in the report, along with earlier anecdotal cases, appear to be associated with relatively mild acne cases, which are primarily influenced by increased sebum production

Subsequent investigations came with a better study design. Li and colleagues [19] performed a double-blind, placebo-controlled, split-face study and investigated the effects of BoNTA on sebum production in 20 volunteers. One side of the face was injected with BoNTA and the other with normal saline. There was a significant decrease in sebum production in the group with facial seborrhea on the BoNTA-treated side compared to the saline-injected side, particularly at four weeks after injection. Interestingly, there was no significant difference between the skin on both sides of the face in the group with dry-to-normal skin. If so, it is plausible to think that facial seborrhea may be more susceptible to cholinergic regulation than normal or dry skin.

Min et al. [32] investigated the dose-response relationship of BoNTA and sebum secretion. In their prospective, randomized, double-blind, dose-comparative clinical trial, 42 female volunteers with forehead rhytides have randomly received an injection of either low dose (10 units) or high dose (20 units) of onabotulinumtoxinA, administered in five standard injections. The sebumeter results illustrated that BoNTA injection significantly reduced sebum excretion at the injection site, with a sebum production gradient surrounding the injection point. Surprisingly, the higher injection did not significantly improve the efficacy of BoNTA.

### 3.5. IncobotulinumtoxinA and AbobotulinumtoxinA

**Figure 3:**
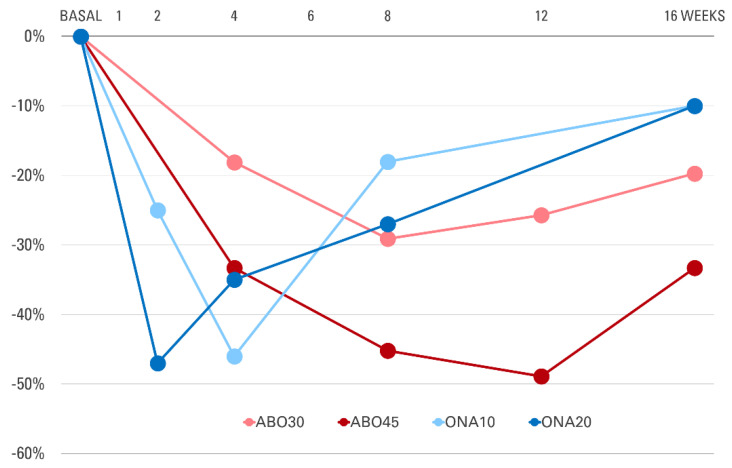
Changes in sebum secretion relative to baseline at each follow-up point after injection of botulinum toxin type A into the forehead. ABO30: abobotulinumtoxinA 30 units; ABO45: abobotulinumtoxinA 45 units; ONA10: onabotulinumtoxinA 10 units; ONA20: onabotulinumtoxinA 20 units. Graph was plotted using the data from [32,34].

A recent study by Park et al. [35] included 20 patients treated with incobotulinumtoxinA (Xeomin; Merz Pharmaceuticals, Frankfurt, Germany) to improve facial skin laxity, sebum secretion, and facial pores. Sebum secretion decreased at one week, and the results were sustained through 12 weeks. All outcomes showed maximum improvement after four weeks. Their findings suggest that not only onabotulinumtoxinA but also incobotulinumtoxinA inhibits sebum production in facial seborrhea. Differences in clinical performance make different BoNTA products not interchangeable. Product comparison between onabotulinumtoxinA and abobotulinumtoxinA (Dysport; Ipsen, Wrexham, UK) is well-described elsewhere. In the literature, we found two clinical trials on the efficacy of abobotulinumtoxinA to reduce sebum production. In their prospective, non-controlled clinical trial, Rose and Goldberg [2] injected abobotulinumtoxinA into the forehead of 25 subjects with facial seborrhea. Intradermal injection of abobotulinumtoxinA resulted in a significant reduction in sebum production at every follow-up point (Figure 3). An average decrease in the sebumeter reading was 80% at a 1-month follow-up, comparable with the sebum reduction rate after oral isotretinoin. In a randomized, double-blinded, placebo-controlled study by Kesty and Goldberg [34], subjects who received 30 or 45 units of abobotulinumtoxinA showed statistically significant decreases of sebumeter readings compared to both the untreated group and the subjects treated with 15 units. Sebum reduction lasted for six months, which is much longer than expected. These results encourage the question of whether abobotulinumtoxinA is more effective than onabotulinumtoxinA in treating facial seborrhea.

### 3.6. Enlarged Facial Pores

Besides a decrease in the sebum secretion, the BoNTA-treated subjects frequently report the associated improvement in the general texture of the skin, highlighted by the “pores shrinkage” [30]. According to Wu [27], the intradermal injection of BoNTA results in an improvement in a decrease in sebum production and a reduction in the number of prominent facial pores. The finding was reaffirmed by Liew [36], demonstrating a reduction of skin pore size at six weeks following intradermal injection of BoNTA in the midface. Considering the sebum output level correlates most significantly with facial pore size [2,37], it is not surprising that BoNTA injection reduces not only the sebum production but also the pore size, as demonstrated in multiple clinical studies [2,19,38,39]. Since nicotinic Ach receptors influence the tissue remodeling activity of dermal fibroblasts [40], a blockade of cholinergic signals by BoNTA may further help diminish prominent facial pores. There are clinical investigations that show the pore improvement efficacy of BoNTA using objective measurements [35,41,42]. However, other factors, such as epidermal regeneration and neocollagenesis after multiple needle pricks, might have contributed to the pore improvement [43].

### 3.7. Limitations of the Procedure

Most clinical trials presented in this review describe an intradermal injection technique, and in only two studies [32,33], the injection depth was intramuscular. Intramuscular injection is generally not recommended when treating facial seborrhea with BoNTA because it may influence the underlying facial expression muscles [2]. Apart from the pain during intradermal injection, BoNTA treatment of facial seborrhea was reported to be generally well-tolerated by the patient and associated with no significant adverse effects such as allergic reaction, facial palsy, or severe paralysis of muscles adjacent to the site of injection. Some patients reported a mild-to-moderate stinging sensation on the injection site, which was temporary and self-limiting [19]. A bruise can occur, but according to the authors’ experience, the risk of bruising can be minimized by keeping the injection depth strictly intradermal. A decrease in the frontalis muscle tension can occur when BoNTA is injected into the inferior parts of the forehead [2]. Intradermal injection of BoNTA in the paranasal skin also can paralyze the lip elevator muscles (zygomaticus minor, levator labii superioris, and levator labii superioris alaeque nasi) and may result in an unnatural facial expression. Disruption of the epidermal barrier may occur, especially when BoNTA injection is combined with the use of topical acne medications. Additional drawbacks to the procedure include the cost of the procedure and a need for repeated treatments. It should also be pointed out that there is no evidence showing the superior efficacy of BoNTA compared to other topical agents that are currently available, for example, topical retinoids.

## 4. Botulinum Neurotoxin for Acne Treatment: Possible Mechanisms

### 4.1. Pathogenesis of Acne

Acne vulgaris is a multifactorial chronic inflammatory disorder of the pilosebaceous unit [44]. Four major factors are known to be involved in the pathogenesis of acne: (1) increased sebum production with altered lipid composition, (2) metagenomic modifications of the bacterial microbiome leading to the *Cutibacterium acnes* (formerly *Propionibacterium acnes*) biofilm formation, (3) abnormal keratinization of the infundibulum and the resulting comedogenesis, and (4) inflammatory cytokine secretion and the infiltration of inflammatory cells into the perifollicular dermis [44,45]. While all four factors are interdependent, it is generally accepted that alterations of the SG and the sebum represent the initial event influencing other pathological processes of acne development. In an acne subject, sebaceous glands and the entire sebaceous follicle are much larger than in non-acne subjects [46]. An early, non-inflammatory acne lesion (a “comedone”) develops when the infundibulum is filled with excessive sebum, and the pore is plugged by the sloughed follicular keratinocytes [45,47]. Considering its inhibitory action on the SG, BoNTA can be a therapeutic modality for treating acne with excessive sebum production.

### 4.2. Acne and Cholinergic Signaling

A growing number of scientific findings indicate that acne may result from an altered cholinergic response of the pilosebaceous unit [44,48]. Several lines of clinical evidence suggest that an exacerbation of acne is related to activation of the stress response through cholinergic signaling [19]. One good example is a positive correlation between cigarette smoking and acne [49,50]. Patients with long-term exposure to nicotine, a major component of tobacco, frequently show an increased sebum secretion, which may cause an acne exacerbation [19]. Human SGs strongly express nAChRa7, meaning that their glandular function is under the regulation of cholinergic signals, at least in part [10,18,19,20,21].

Not only the SG but also the follicular epithelium is under the control of the cholinergic system. There is ample evidence that ACh and nicotine play essential roles in keratinocyte adhesion, migration, differentiation, and apoptosis [12,51] and may participate in acne pathogenesis by promoting infundibular epithelial hyperplasia and thus follicular plugging [19]. Data show that nicotine and the non-neuronal cholinergic system play a causative role in the pathogenesis of hidradenitis suppurativa, a chronic inflammatory disorder of the pilosebaceous unit, by stimulating the sebum secretion and the proliferation of the follicular epithelium [48]. Ach activates the adhesion and proliferation of keratinocytes by reacting with cholinergic receptors on their cell surfaces [12], implying that BoNTA may inhibit adhesion of the infundibular keratinocytes, which enables better clearance of hair follicle opening and prevents the formation of comedones.

**Figure 4:**
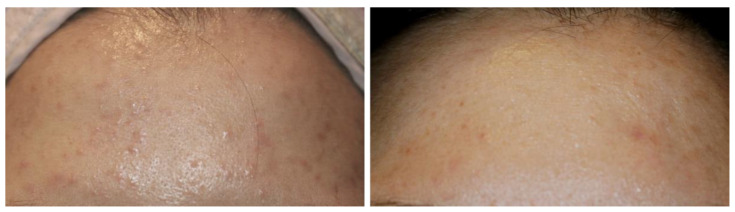
A young Korean female with facial seborrhea and acne on the forehead. Two weeks after intradermal injection of 12 units of onabotulinumtoxinA, a significant improvement was observed.

Several clinical observations imply the role of BoNTA in the treatment of acne. A neurologic report describes a marked improvement of facial acne following BoNTA injections to treat facial tics in patients with Tourette syndrome [52]. In the report, clearing of perinasal acne was observed 1–2 weeks after injecting 20 to 25 units of onabotulinumtoxinA into the paranasal facial expression muscles. The clinical improvement lasted about four months. In a controlled study of BoNTA for the treatment of facial rhytides, acne developed at a lower rate in the treatment group than in the placebo group [53]. Intradermal injection of BoNTA is particularly effective for treating acne on the forehead, associated with excess sebum secretion (Figure 4).

### 4.3. Catecholamines

Changes in acne severity correlate highly with increasing emotional stress [54]. The most widely accepted explanation may be the role of adrenergic signaling in the functional regulation of SGs [55]. Upon a stress response, our body triggers the sympathetic–adrenomedullary axis to release catecholamines (epinephrine, norepinephrine, and dopamine), to which human SGs respond [55,56]. Sebocytes treated with norepinephrine or epinephrine showed an increase in intracellular lipid accumulation in a dose-dependent manner. Norepinephrine and epinephrine in sebocytes augment the transcription of the sebaceous gland differentiation markers [57]. Moreover, catecholamines increase biofilm formation and stimulate sebocyte lipid synthesis by *C. acnes* [58]. Botulinum neurotoxins modulate sympathetic neurons during intense activation by physiological or pathophysiological stimuli, as Zhou et al. [59] showed that BoNTA inhibits adrenergic response in a dose-dependent manner by cleaving SNAP-25 in sympathetic neurons. Data from an animal study showed that BoNTA induces prolonged sympathetic block when placed on sympathetic ganglia [60]. A clinical trial in patients with complex regional pain syndrome also demonstrated a sympathetic inhibition by BoNTA [61]. Several studies [62,63,64] highly suggest an inhibitory effect of botulinum neurotoxins on the secretion of catecholamines, providing further rationale for the use of BoNTA in acne treatment. The detailed mechanism of BoNTA affecting the sympathetic pathway needs to be further investigated.

### 4.4. Mediators of Inflammation in Acne

A frequent exacerbation of acne during periods of emotional stress is also related to neuropeptide secretion and the resulting inflammation. Among many neurogenic peptides, substance P is well known to contribute to the onset and the exacerbation of inflammation in acne. In vitro study results suggest that substance P stimulates the lipogenesis in the SGs of acne patients, followed by the proliferation of *C. acnes* and the provocation of inflammatory reactions [65]. Knowing that BoNTA prevents the release of substance P both in vitro and in vivo [66], its anti-inflammatory properties may help resolve inflammation in acne and other disorders of the hair follicle [67]. Findings from a series of investigations [68,69] also suggest that BoNTA may benefit acne patients by blocking ACh and inhibiting the release of several neurogenic transmitters.

When *C. acnes* and other bacterial species act on the SG lipid to stimulate the release of cytokines, sebaceous follicles release various inflammatory mediators. Arachidonic acid is one of the crucial inflammatory mediators in acne pathogenesis and exerts diverse roles in the development of acne inflammation [70,71]. BoNTA exerts an anti-inflammatory role in acne by lowering the intraterminal arachidonic acid level [72]. Interestingly, arachidonic acid is known to induce a release of ACh at cholinergic nerve terminals [72], which BoNTA can best suppress. In an animal study [73], BoNTA also inhibited the expression of cyclooxygenase-2, a key enzyme for cytokine-mediated acne inflammation [70]. Inflammatory acne lesions show an increased number and activity of mast cells in their SGs [65,74]. BoNTA inhibits mast cell activity by affecting soluble *N*-ethylmaleimide-sensitive-factor attachment protein receptor (SNARE) proteins, including synaptosomal-associated protein-25 (SNAP-25) and vesicle-associated membrane protein (VAMP) [75]. BoNTA also inhibits signaling of the transient receptor potential vanilloid subtype 1 (TRPV1) [76], which is a regulator of human sebocyte activities and the disease process of acne [77,78]. Of note, TRPV1 is an important pathogenetic factor in the inflammatory process of pain syndromes, chronic itch, and rosacea, all known indications of BoNTA.

Collectively, all these findings suggest that a strong involvement of inflammatory mediators in the SG can be a target of acne treatment using BoNTA.

## 5. Conclusions

**Figure 5:**
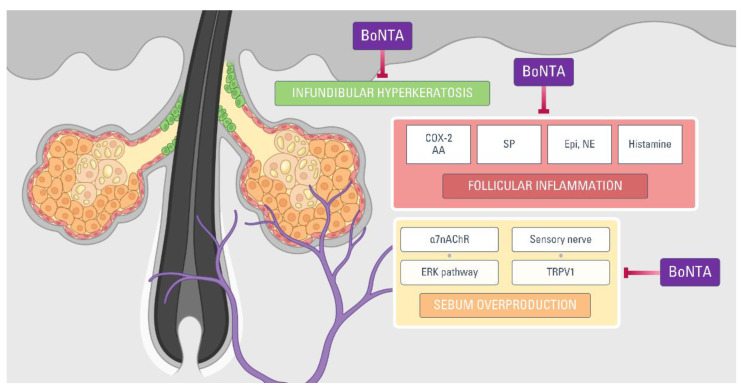
Pathogenetic factors of acne that can be inhibited by botulinum neurotoxin type A. α7nAChR: alpha 7 nicotinic acetylcholine receptor; AA: arachidonic acid; BoNTA: botulinum neurotoxin type A; COX-2: cyclooxygenase-2; Epi: epinephrine; NE: norepinephrine; SP: substance P; TRPV1: transient receptor potential vanilloid subtype 1.

BoNTA affects cholinergic transmission and other neurotransmitter pathways of SG activities. Data from in vitro and in vivo studies indicate that BoNTA can be a potential tool to treat facial seborrhea and acne, closely related to abnormal sebaceous gland activities (Figure 5). A review of clinical trials revealed that intradermal injection of BoNTA is an effective and safe treatment of excessive sebum secretion and prominent facial pores. Since BoNTA is not labeled for acne treatment, it should be kept as a secondary option in acne management until more clinical trials confirm these promising results. Further research is recommended to present more robust evidence for the functional role of BoNTA in SG biology.

## Figures and Tables

**Table 1:** Quantitative studies on the effect botulinum neurotoxins on sebum secretion in patients with facial seborrhea.

First Author	Product	Injection	*n*	Findings (As Described in the Paper)
Min [32]	ONA	IM	42	A significant decrease in sebum production A sebum gradient surrounding the injection point No difference in the efficacy between the 10 units group and the 20 units group Recovery of the sebum production at the 16-week follow-up
Kondrateva [33]	ONA	IM	15	Maximum decrease in sebum production at the 4-week follow-up A return-to-baseline at 16 weeks after injection
Hathout [30]	ONA	ID	20	A significant decrease in sebum production Patient satisfaction up to 3 months Associated improvement in the skin tone and facial pores No influence of age, gender, or skin types
Shirshakova [31]	ONA	ID	12	A decrease of skin surface sebum amount at 1 week and 2 weeks after injection
Li [19]	MTX	ID	20	A marked decrease in sebum production on the BoNTA-treated side in facial seborrhea group
Rose [2]	ABO	ID	25	A significantly lower sebum production at 1, 4, 8, and 12 weeks after injection
Kesty [34]	ABO	ID	50	A significant decrease in sebum production in the treatment groups 30 units and 45 units The effect lasted for 6 months after injection

ABO: abobotulinumtoxinA (Dysport; Ipsen, Wrexham, UK); ID: intradermal injection; IM: intramuscular injection; MTX: Meditoxin (Medy-Tox Inc., Seoul, Korea); ONA: onabotulinumtoxinA (Botox; Allergan, CA, USA).

## Data Availability

Not applicable.
